# Construction of a ceRNA network to reveal a vascular invasion associated prognostic model in hepatocellular carcinoma

**DOI:** 10.1515/med-2023-0795

**Published:** 2023-09-13

**Authors:** Yun Liu, Lu Yang, Mengsi Yu, Fen Huang, Jiangzheng Zeng, Yanda Lu, Changcheng Yang

**Affiliations:** Department of Medical Oncology, The First Affiliated Hospital of Hainan Medical University, Haikou, Hainan 570102, P.R. China; Department of Clinical Laboratory, The First Affiliated Hospital of Xinjiang Medical University, Urumqi, Xinjiang 830054, P.R. China; Department of Medical Oncology, The First Affiliated Hospital of Hainan Medical University, 31 Longhua Road, Haikou, Hainan 570102, P.R. China

**Keywords:** HCC, vascular invasion, circRNA, ceRNA network, prognosis

## Abstract

The aim of this study is to explore the prognostic value of vascular invasion (VI) in hepatocellular carcinoma (HCC) by searching for competing endogenous RNAs (ceRNA) network and constructing a new prognostic model for HCC. The differentially expressed genes (DEGs) between HCC and normal tissues were identified from GEO and TCGA. StarBase and miRanda prediction tools were applied to construct a circRNA-miRNA-mRNA network. The DEGs between HCC with and without VI were also identified. Then, the hub genes were screened to build a prognostic risk score model through the method of least absolute shrinkage and selection operator. The prognostic ability of the model was assessed using the Kaplan−Meier method and Cox regression analysis. In result, there were 221 up-regulated and 47 down-regulated differentially expressed circRNAs (DEcircRNAs) in HCC compared with normal tissue. A circRNA-related ceRNA network was established, containing 11 DEcircRNAs, 12 DEmiRNAs, and 161 DEmRNAs. Meanwhile, another DEG analysis revealed 625 up-regulated and 123 down-regulated DEGs between HCC with and without VI, and then a protein–protein interaction (PPI) network was built based on 122 VI-related DEGs. From the intersection of DEGs within the PPI and ceRNA networks, we obtained seven hub genes to build a novel prognostic risk score model. HCC patients with high-risk scores had shorter survival time and presented more advanced T/N/M stages as well as VI occurrence. In conclusion a novel prognostic model based on seven VI-associated DEGs within a circRNA-related ceRNA network was constructed in this study, with great ability to predict the outcome of HCC patients.

## 1 Introduction

Hepatocellular carcinoma (HCC) is one of the most common malignant tumors and the fourth leading cause of cancer-related deaths worldwide. The highest incidence rates of HCC in the world are reported in Asia and Africa. Although Mongolia has the highest incidence (93.7 per 100,000), China has the largest number of HCC patients, due to both a relatively high incidence (18.3 per 100,000) and the world’s largest population (1.4 billion persons). Though the great advances in diagnosis and therapy, the prognosis of HCC patients remains poor, with mortalities approximating incidence rates worldwide [1,2,3]. The average 5 year survival rate of HCC patients in the US was 19.6%, but it was only 2.5% for advanced HCC patients, and the poor prognosis was closely related to tumor metastasis [4]. Vessel is an important pathway for cancer cells to invade other organs, and angiogenesis is essential for tumor growth and metastasis. After the cancer cells penetrate the microvessels, they mainly contact the basement membrane and bind to matrix proteins through special membrane receptors, such as certain integrin receptors on the surface of cancer cells binding to laminin in the matrix, thus entering the metastatic tissues. Vascular invasion (VI) includes macrovascular invasion or microvascular invasion, and represents the aggressive nature of the spread of the tumor cells. Invasion of the hepatic venous tributaries can cause systemic metastasis, while invasion of portal venous may result in intrahepatic spread of the tumor cells [5]. Evidence showed that VI was an independent prognostic indicator for HCC [6]. The presence of VI was an unfavorable prognostic factor of HCC recurrence and overall survival (OS) [7]. Currently, the specific mechanism of VI and metastasis of HCC is still not well understood. Thus, to better understand the molecular mechanism underlying VI of HCC is vital for assessing the risk of HCC metastasis and patients’ survival [8]. In addition, by studying the mechanism of VI, molecularly targeted therapies can be used to achieve anticancer effects, such as accurate and targeted attack on tumor cells, inhibition of angiogenesis and metastasis of cancer cells, and reversal of multidrug resistance.

circRNAs are a class of non-coding RNAs that are widely expressed in mammalian cells. circRNAs were reported to regulate many cellular processes including tumor initiation and progression [9]. Recently, a large number of circRNAs have been demonstrated to play important roles in HCC development via involvement in the competing endogenous RNA (ceRNA) network. For example, circ_0067835 was found to be elevated in HCC and could promote cell proliferation and metastasis via miR-1236-3p/Twist2 axis [10]. circRASSF5 was reported as a tumor suppressor in HCC, which could competitively sponge miR-331-3p and thus enhance the tumor inhibitory effect of PH domain and leucine rich repeat protein phosphatase [11]. circ0003998 was shown to act as a ceRNA of miR-143-3p to relieve the repressive effect on FOSL2, an EMT-related stimulator, thus promoting HCC metastasis [12].

Currently, TCGA and GEO databases were widely used to screen differentially expressed genes (DEGs) to build prognostic signatures for assessing the survival of HCC patients. One of the important screening strategies is to select biological hub genes within the ceRNA network. For instance, Chen et al. [13] constructed a lncRNA-related ceRNA regulatory network for HCC using DEGs from TCGA database, and then 11 lncRNAs within the ceRNA network were selected to build a prognostic signature. The multivariate Cox regression analysis showed that the prognostic signature could be an independent indicator for HCC patients’ survival. Zhang et al. [14] reported a differential lncRNA-miRNA-mRNA regulatory network in HCC based on TCGA-LIHC data and three key lncRNAs were eventually screened to construct a prognostic signature for OS. Similarly, Huang et al. [15] constructed a ceRNA network comprising 44 DEmRNAs, 7 DElncRNAs, and 20 DEmiRNAs in hepatitis B virus (HBV)-related HCC, and then established a 7-lncRNA signature, which was finally verified as a potential prognostic predictor for HBV-related HCC patients. Notably, as VI has been identified to be a prognostic index for HCC patients’ survival, a VI-related ceRNA network and a 8-lncRNA prognostic model were subsequently constructed by Tao et al. and time-dependent ROC analysis verified the utility of the model to predict the clinical outcomes of HCC patients [16]. Currently, several circRNA-related ceRNA networks were constructed for HCC. However, there was no prognostic model for HCC built based on the hub genes in relation to VI and circRNA-related ceRNA network simultaneously so far.

Here we sought to establish a novel prognostic signature for HCC by selecting VI-related hub genes within the circRNA-related ceRNA network. In this study, we first explored the DEGs between HCC and normal tissue through GEO and TCGA databases. Then, we constructed a circRNA-miRNA-mRNA network and investigated the biological functions of these HCC-related DEGs. Additionally, the DEGs between HCC with and without VI were also screened to build a protein–protein interaction (PPI) network. After making the intersection of DEGs within PPI and ceRNA networks, we obtained seven hub genes to construct a novel prognostic risk score model. The prognostic value of the model for HCC patients’ survival was further investigated. Finally, the immune cell infiltration in HCC was assessed according to the risk scores from the prognostic model.

## 2 Methods and materials

### 2.1 Data acquisition and processing

We downloaded the circRNA expression dataset GSE94508 [17] and GSE97332[18] containing human HCC and adjacent normal tissues from GEO (https://www.ncbi.nlm.nih.gov/geo/) database. Two sets of data are from platform GPL19978 and our search terms were HCC and circRNA. There are 10 samples in GSE94508, which are divided into 5 tumor tissues and 5 tumor adjacent normal tissues, and 14 samples in GSE97332, consisting of 7 tumor tissues and 7 tumor adjacent normal tissues. The primary data were processed by background correction and quantile normalization, and the integrated circRNA expression data were obtained after batch effect removal using R package sva [19]. The ComBat function in R packet sva [19] was used to integrate multiple data and remove the batch effect. We used TCGAbiolinks package [20] to download HCC (Liver hepatocellular carcinoma, TCGA-LIHC)-related gene expression data from the TCGA database (https://portal.gdc.cancer.gov/), which contained 371 HCC tissues and 50 tumor adjacent normal tissues. Meanwhile, TCGAbiolinks package [20] was used to obtain the corresponding clinicopathological survival information of 371 tumor samples, including age, survival status, follow-up time, VI, stage, and so on. In addition, the gene expression data of normal liver tissue (GTEX-Liver) were downloaded from GTEX database, which contained 110 normal samples. Two datasets of TCGA-LIHC and GTEX-Liver were combined to extract the expression data of common genes, and the batch effect between different datasets was corrected by ComBat function in R package sva [19]. The combined data included 371 HCC tumor samples and 160 control samples. Gene expression data included miRNA and mRNA expression levels of each patient, and clinical information included age, sex, pathological stage, VI, survival status, and total survival time. Only patients with complete survival information and gene expression data were included in this study. Finally, a total of 358 patients were selected for further analysis.

### 2.2 Screening of DEGs

In order to assess the difference of gene expression between HCC and normal tissue, R packet limma [21] was used to analyze the difference among different groups. |logFC| > 1 and *P* value < 0.05 were set as thresholds for differentially expressed circRNAs (DEcircRNAs), where circRNAs with logFC > 1 were considered significantly up-regulated, and circRNAs with logFC < −1 were considered down-regulated. For differentially expressed mRNAs (DEmRNAs), we set the threshold value as |logFC| > 2 and *P* value < 0.05, where DEmRNAs with logFC < −2 were down-regulated and DEmRNAs with logFC > 2 were up-regulated. Similarly, |logFC| > 1 and *P* value < 0.05 were used to identify differentially expressed miRNAs (DEmiRNAs). The volcano map was used to show the up and down-regulated DEGs, and pheatmap R-package [22] was used to draw the heat map of these DEGs in all samples.

### 2.3 Construction of a ceRNA network

To better understand the effect of circRNAs on the progression of HCC, a ceRNA network was built based on DEcircRNAs, DEmiRNAs, and DEmRNAs. The human sequences of DEcircRNAs and DEmiRNAs were downloaded from circBase (http://www.circbase.org/) and miRBase (version 21; http://www.mirbase.org/) databases, respectively. The miRanda prediction tool was used to predict the interaction between DEcircRNAs and DEmiRNAs. In addition, the potential mRNAs targeted by DEmiRNAs were obtained from StarBase (http://starbase.sysu.edu.cn/) database, which provided prediction results from seven prediction programs (TargetScan, microT, miRmap, picTar, RNA22, PITA, and miRanda). If the interaction between miRNA and mRNA was predicted in not less than four programs, it was selected for next analysis. Then, we overlapped the target mRNAs with the DEmRNAs mentioned above. The nodes that could not form circRNA-miRNA-mRNA interaction relationship were removed, and finally a ceRNA network was established and visualized by the Cytoscape software (version 3.7.0/www.cytoscape.org).

### 2.4 Functional enrichment analysis

Gene Ontology (GO) functional annotation analysis is a common method for large-scale functional enrichment of genes [23], including biological processes (BPs), molecular functions (MFs), and cellular components (CC). Kyoto Encyclopedia of Genes and Genomes (KEGG) is a widely used database for storing information about genomes, biological pathways, and drugs [24]. In order to study the BPs that the ceRNA network might participate in, we chose the DEmRNAs within the ceRNA network for GO functional annotation analysis and KEGG pathway enrichment analysis by using clusterProfiler R software package [25]. *P* value < 0.05 was considered statistically significant.

### 2.5 Gene expression difference between HCC with and without VI

According to the clinical information of patients with HCC, we divided patients into two groups according to HCC with or without VI; 236 patients with HCC marked “None” without VI, and 122 patients with HCC labeled “Micro” and “Macro” with VI. In order to explore the difference between HCC with and without VI, limma R-packet was used to analyze the DEGs between the two groups. |logFC| > 1 and *P*adj < 0.05 were set as the threshold of DEGs. The genes of logFC > 1 and *P*adj < 0.05 were regarded as the up-regulated DEGs, and the genes of logFC < −1 and *P*adj < 0.05 were down-regulated. The volcano map was used to show the up-regulated and down-regulated DEGs, and the pheatmap R-package drew the heat map of these DEGs in all samples.

### 2.6 Construction of PPI network

PPI network is composed of individual proteins through the interaction between each other, which participates in various life processes such as biological signal transmission, gene expression regulation, and cell cycle regulation. PPI analysis is very important for elucidating the molecular mechanism of key cellular activities in carcinogenesis. Based on the DEGs between HCC with and without VI, the STRING database (https://string-db.org/) was used to evaluate the PPI information and further construct the PPI network [20]. Taking the interaction score of 0.4 as the cut-off value, the PPI network was visualized by cytoscape software.

### 2.7 Construction of prognostic model and analysis of prognosis

Based on the intersection of DEGs within the PPI and ceRNA network mentioned above, we constructed a prognostic risk score model through the method of least absolute shrinkage and selection operator (LASSO) to realize the risk stratification of HCC patients. LASSO regression is a commonly used variable selection method when fitting high-dimensional generalized linear models. The glmnet package [26] in R was used to execute the LASSO algorithm. The risk scoring model was established by combining the regression coefficient with the corresponding gene expression value. After calculating the risk score, taking the median risk score as the cut-off point, the patients in each cohort were divided into low- and high-risk group accordingly. Univariate and multivariate Cox analyses were used to analyze the ability of risk score in combination with clinicopathological features to predict the OS. Then, the risk score model and clinicopathological parameters were used for further analysis through the clinical predictive Nomogram, which was constructed by rms R-packet [27]. Decision curve analysis (DCA) was performed, and clinical impact curves were drawn to evaluate whether the model-based decisions were beneficial to patients.

### 2.8 Difference of immune cell infiltration between high- and low-risk groups

The immune microenvironment is mainly composed of immune cells, inflammatory cells, fibroblasts, endothelial cells, bone marrow-derived cells, extracellular matrix, cytokines, and chemokines, which is a comprehensive system. The analysis of immune cells infiltration in the clinical samples is of great importance to discern the mechanisms underlying cancer progression and predict prognosis. Single sample gene cluster enrichment analysis (ssGSEA) is an extension of GSEA method. ssGSEA algorithm was used to calculate the content of 28 kinds of immune cells in high- and low-risk groups [28]. The composition of immune cells in high and low risk groups was visualized by box map.

### 2.9 Statistical analysis

All statistical analyses were carried out in R language (https://www.r-project.org version 4.0.2). For the comparison of continuous variables in two groups, the statistical significance of normal distribution variables was estimated by independent *t*-test, the differences between non-normal distribution variables were analyzed by Wilcoxon rank sum test, and the differences between multiple groups of independent variables were analyzed by Kruskal−Wallis Test. The Kaplan−Meier (KM) method was used to evaluate the difference in survival time of patients with HCC, and the logarithmic rank test was used to determine the statistical significance of the observed differences between distinct groups. The hazard ratio and 95% confidence interval were calculated based on Cox regression analysis. All the statistical *P* values were bilateral, *P* < 0.05 was considered statistically significant.


**Ethics approval and consent to participate:** TCGA and GEO both are public databases. The patients involved in the database have given their approval in the original studies. Users can download relevant data for research and publish relevant articles. Our study is based on these open-source data, so there are no ethical issues.

## 3 Results

### 3.1 Identification of DEGs

**Figure 1:**
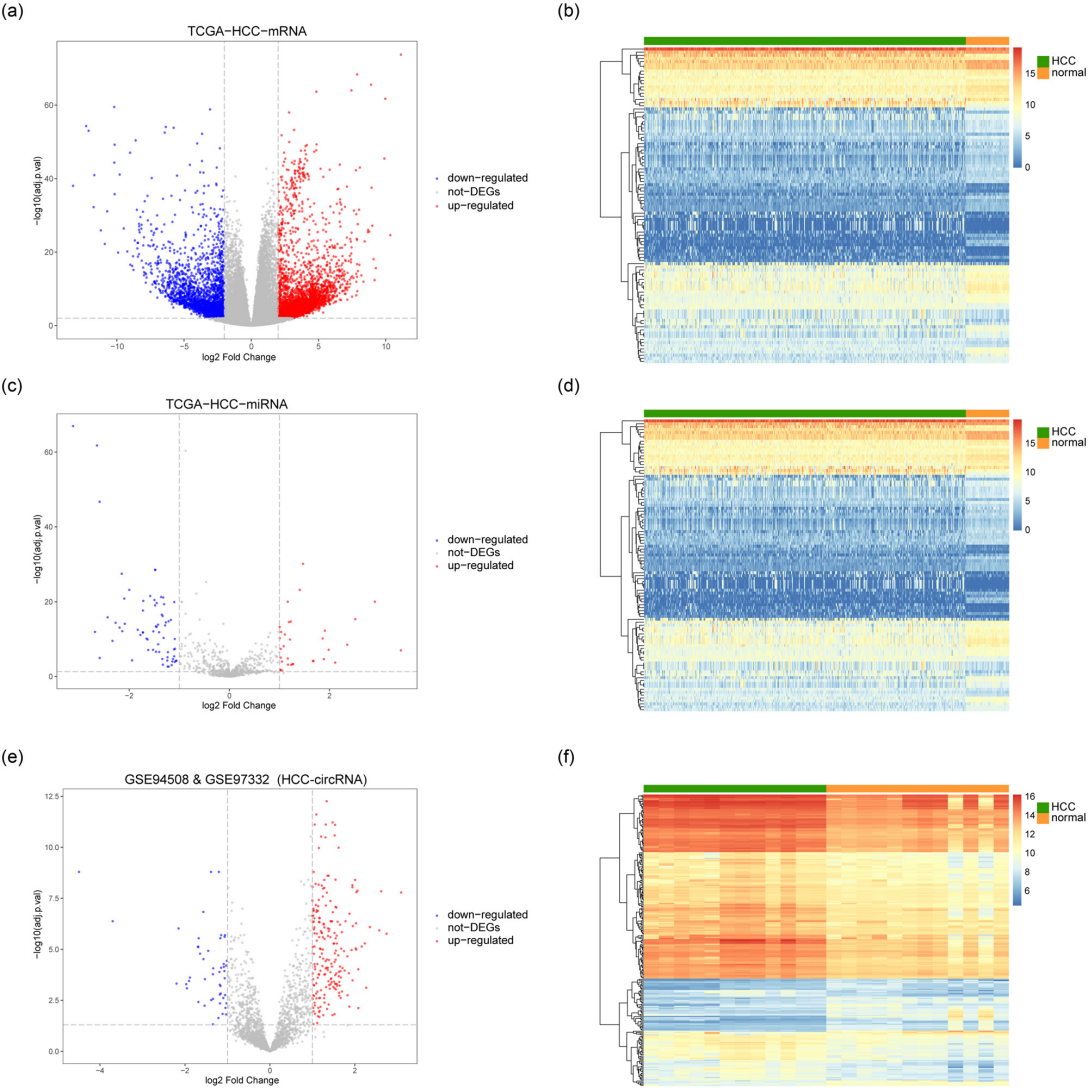
Screening of DEGs between HCC and normal tissue. (a) and (b) Identification of DEmRNA. (c) and (d) Identification of DEmiRNAs. (e) and (f) Identification of DEcircRNAs. Abscissa was log2 FoldChange, ordinate was log10 (adjust *P*-value), red nodes represent up-regulated DEGs, blue nodes represent down-regulated DEGs, and grey nodes represent non-DEGs.

**Figure A1:**
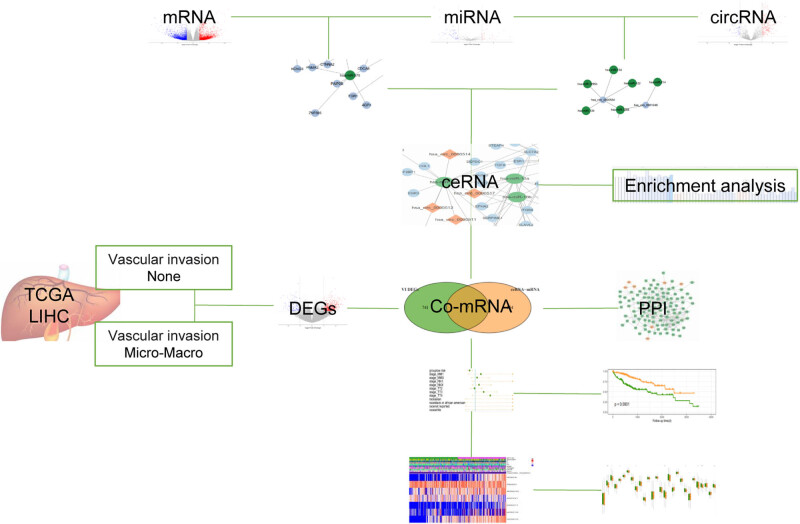
The schematic diagram showed the analysis strategy of our study.

The schematic diagram of our analysis strategy is shown in Figure A1. To analyze the difference of gene expression between HCC and normal tissue, we first analyzed the mRNA expression data. We used limma differential analysis to get 6,674 DEmRNAs, including 4,446 up-regulated DEmRNAs and 2,228 down-regulated DEmRNAs. Using DEmRNAs and data grouping information to draw a classification heat map, DEmRNAs could well distinguish HCC from normal tissue (Figure 1a and b). For miRNA expression data, we used limma differential analysis to get 100 DEmiRNAs, including 33 up-regulated DEmiRNAs and 67 down-regulated DEmiRNAs. Using DEmiRNAs and data grouping information to draw a classification heat map, DEmiRNAs could well differentiate HCC from normal tissue (Figure 1c and d). For the integrated circRNA expression data, we used limma difference analysis to get 211 up-regulated DEcircRNAs and 47 down-regulated DEcircRNAs. Using DEcircRNAs and data grouping information to draw a classification heat map, DEcircRNAs could also well discriminate HCC from normal tissue (Figure 1e and f).

### 3.2 Construction of the ceRNA network based on HCC-related DEGs

**Figure 2:**
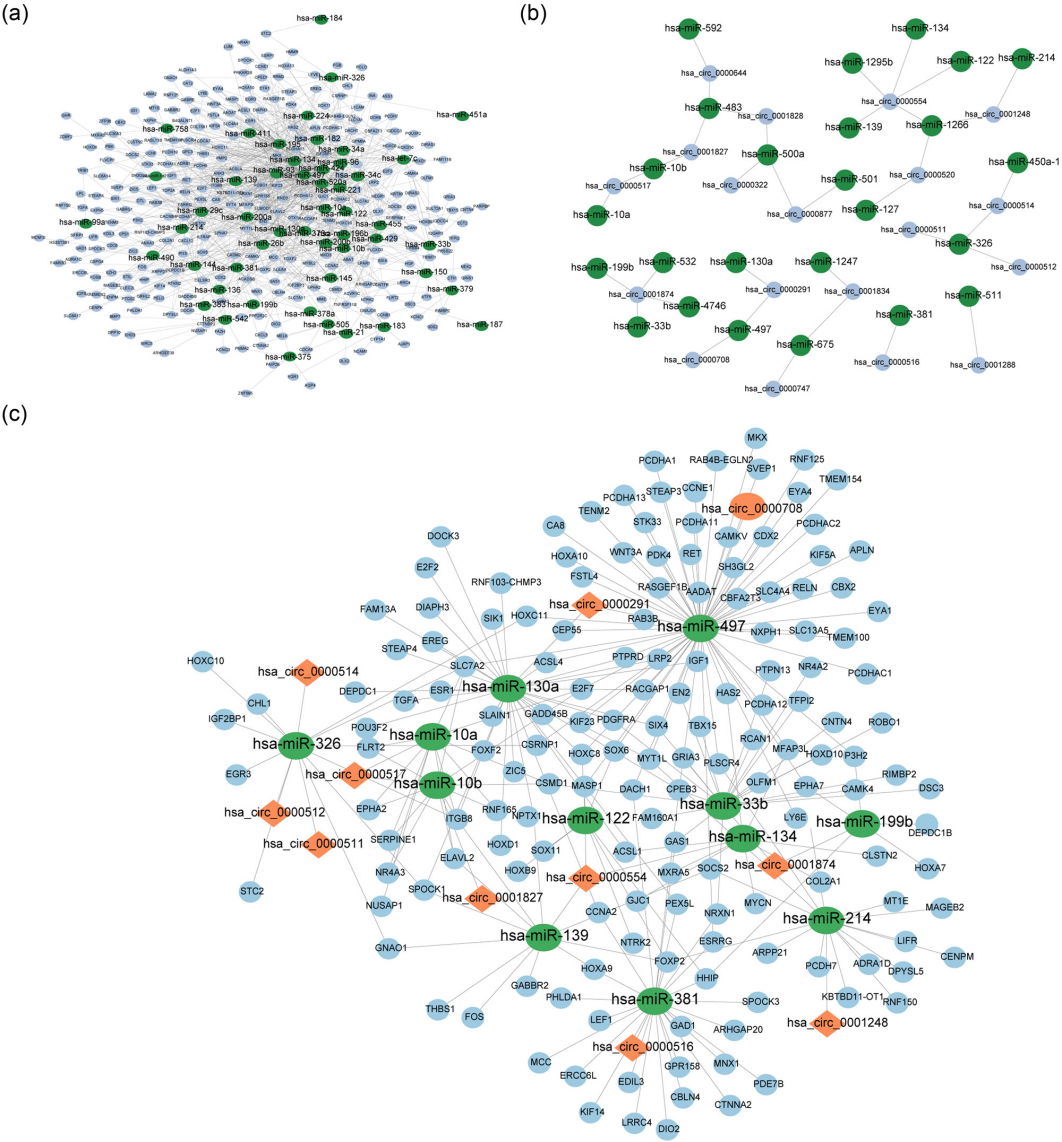
Construction of the ceRNA network. (a) miRNA-mRNA interaction network, green node represents miRNA and blue node represents mRNA. (b) circRNA-miRNA interaction network, green node represents miRNA and blue node represents circRNA. (c) circRNA-miRNA-mRNA interaction network, green node represents miRNA, orange node represents circRNA, and blue node represents mRNA.

We used the StarBase database to identify the DEmRNAs targeted by DEmiRNAs. A total of 1,059 miRNA-mRNA interactions were predicted, including 51 DEmiRNAs and 316 DEmRNAs (Figure 2a). Using the miRanda prediction tool, 36 circRNA-miRNA interactions were predicted, based on 19 DEcircRNAs and 25 DEmiRNAs (Figure 2b). After integrating the circRNA-miRNA interaction with the miRNA-mRNA interaction and removing the nodes that cannot form circRNA-miRNA-mRNA interaction, a novel HCC-related ceRNA network was established. The network consisted of 11 DEcircRNAs, 12 DEmiRNAs, and 161 DEmRNAs (Figure 2c).

### 3.3 Functional enrichment analysis of DEmRNAs

**Figure 3:**
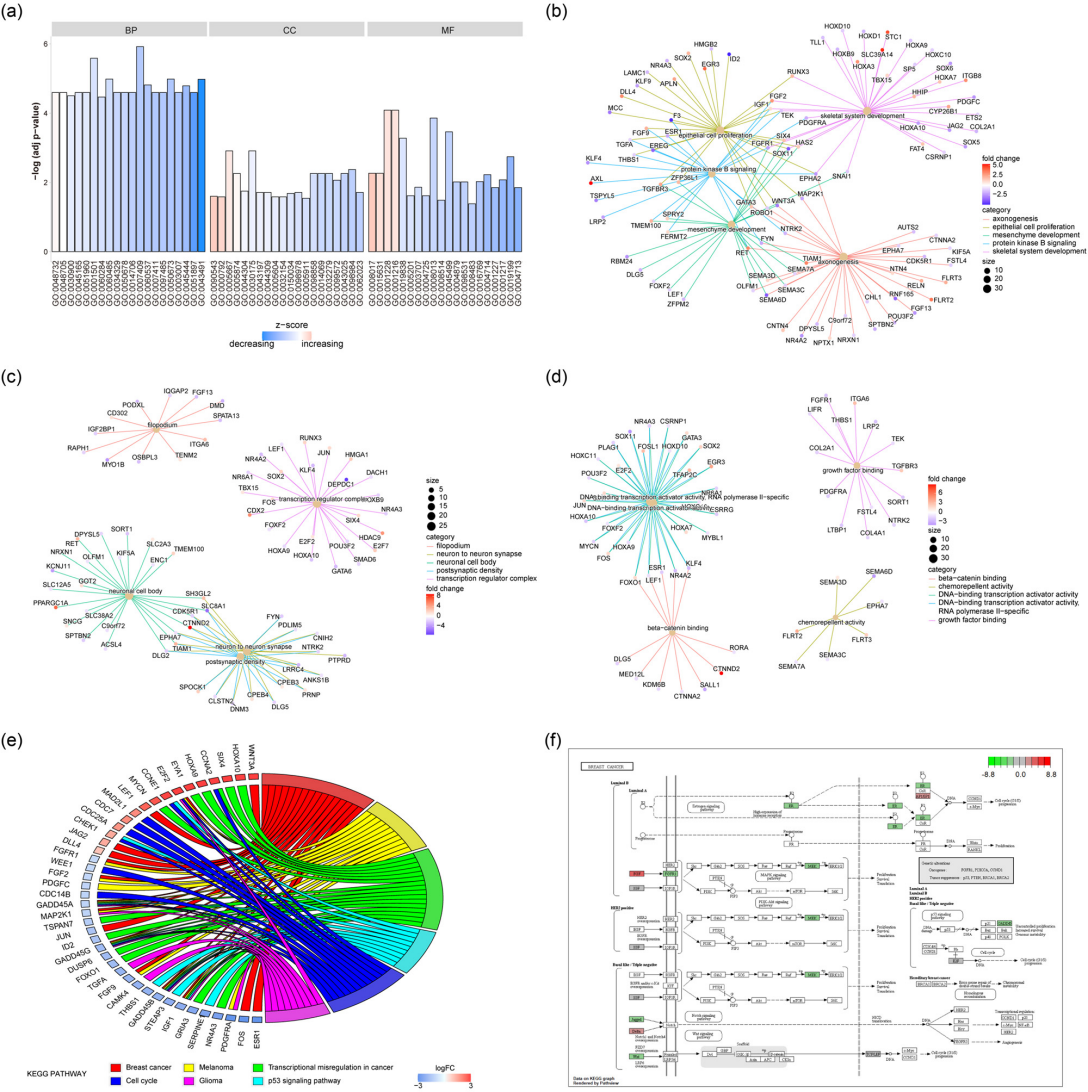
GO and KEGG enrichment analysis. (a) GO function enrichment analysis, ordinate was log10 (*P* value), abscissa was GO terms, node color represented z-score. (b)−(d) The top five items of BP, CC, and MF showed that the node size indicated the number of genes contained in the current GO term, the color of the line represents different GO terms, and the node color represents the FC of the gene. (e) KEGG pathway enrichment analysis showed that the node color represents the FC of the gene, and the line color represents different KEGG pathways. (f) Significantly enriched KEGG pathway, hsa05224: Breast cancer.

**Figure A2:**
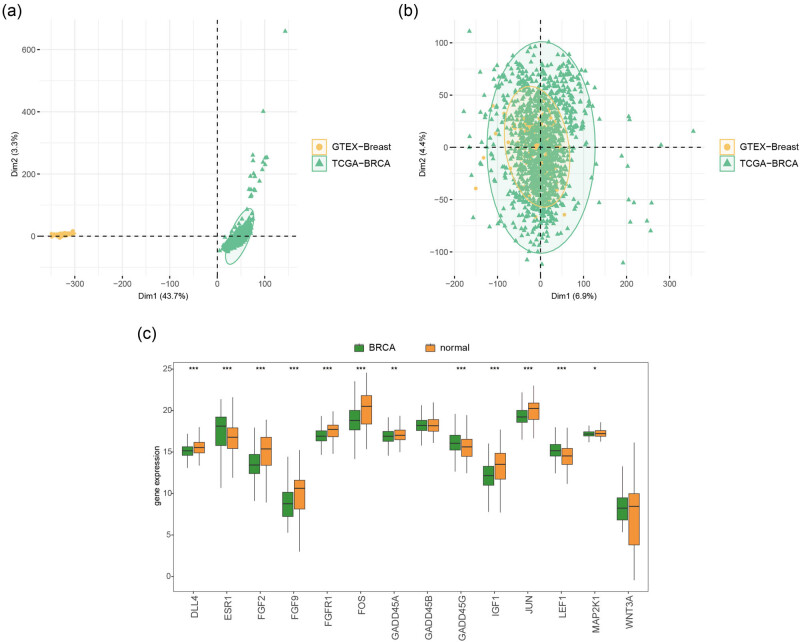
(a) There are obvious batch effects in TCG A-BRCA and GTEX- Breast. (b) TCG A-BRCA and GTEX- Breast data are integrated and batch effects are removed for subsequent analysis. (c) Of the 14 differentially expressed genes in hepatocellular carcinoma, 12 were also significantly differentially expressed in breast cancer, * indicating *P* < 0.05, **indicating *P* < 0.01, *** indicating *P* < 0.001.

In order to explore the biological functions of these HCC-related DEmRNAs, we performed the functional enrichment analysis (Figure 3a and Tables 1 and 2) HCC-related DEmRNAs were mainly enriched in BPs associated with axonogenesis, skeletal system development, mesenchyme development, epithelial cell proliferation, and protein kinase B signaling (Figure 3b). At the same time, it was enriched in CCs such as filopodium, transcription regulator complex, neuron to neuron synapse, neuronal cell body, and postsynaptic density (Figure 3c) and MFs such as DNA-binding transcription activator activity, RNA polymerase II-specificity, DNA-binding transcription activator activity, beta-catenin binding, chemorepellent activity, and growth factor binding (Figure 3d). Then, the pathway enrichment analysis was carried out, and the results showed that HCC-related DEmRNAs were enriched in biological pathways such as breast cancer, melanoma, transcriptional mis-regulation in cancer, p53 signaling pathway, cell cycle, glioma, and so on (Figure 3e). Figure 3f shows the most significant enrichment pathway: hsa05224: breast cancer (Figure 3F). Here we found that it was intriguing to observe the enrichment of a pathway for a different cancer type. Therefore, we compared the DEGs between breast cancer and HCC from TCGA database, and found that there were commonalities between these two cancer types (Figure A2).

**Table 1:** GO analysis of DEmRNAs

Ontology	ID	Description	*P*.adjust
BP	GO:0007409	Axonogenesis	2.82 × 10^−10^
BP	GO:0001501	Skeletal system development	1.22 × 10^−9^
BP	GO:0060485	Mesenchyme development	7.40 × 10^−9^
BP	GO:0050673	Epithelial cell proliferation	9.80 × 10^−9^
BP	GO:0043491	Protein kinase B signaling	1.24 × 10^−8^
BP	GO:0060537	Muscle tissue development	2.16 × 10^−8^
BP	GO:0045444	Fat cell differentiation	2.67 × 10^−8^
BP	GO:0045165	Cell fate commitment	5.34 × 10^−8^
BP	GO:0048732	Gland development	5.84 × 10^−8^
CC	GO:0030175	Filopodium	3.31 × 10^−6^
CC	GO:0005667	Transcription regulator complex	6.30 × 10^−6^
CC	GO:0098984	Neuron to neuron synapse	3.24 × 10^−6^
CC	GO:0043025	Neuronal cell body	7.33 × 10^−5^
CC	GO:0014069	Postsynaptic density	8.89 × 10^−5^
CC	GO:0005874	Microtubule	9.02 × 10^−5^
CC	GO:0032279	Asymmetric synapse	0.00010944
CC	GO:0098858	Actin-based cell projection	0.000112462
CC	GO:0099572	Postsynaptic specialization	0.00019835
CC	GO:0044304	Main axon	0.000456943
MF	GO:0001228	DNA-binding transcription activator activity, RNA polymerase II-specific	2.10 × 10^−7^
MF	GO:0001216	DNA-binding transcription activator activity	2.54 × 10^−7^
MF	GO:0008013	Beta-catenin binding	6.49 × 10^−7^
MF	GO:0045499	Chemorepellent activity	2.16 × 10^−6^
MF	GO:0019838	Growth factor binding	4.12 × 10^−6^
MF	GO:0019199	Transmembrane receptor protein kinase activity	1.69 × 10^−5^
MF	GO:0015631	Tubulin binding	6.02 × 10^−5^
MF	GO:0008017	Microtubule binding	6.79 × 10^−5^
MF	GO:0004714	Transmembrane receptor protein tyrosine kinase activity	8.36 × 10^−5^
MF	GO:0001217	DNA-binding transcription repressor activity	0.000131863

**Table 2:** KEGG analysis of DEmRNAs

Ontology	ID	Description	*P*.adjust
KEGG_PATHWAY	hsa05224	Breast cancer	2.40 × 10^−6^
KEGG_PATHWAY	hsa05218	Melanoma	3.56 × 10^−6^
KEGG_PATHWAY	hsa05202	Transcriptional mis-regulation in cancer	7.51 × 10^−5^
KEGG_PATHWAY	hsa04115	p53 signaling pathway	0.000157097
KEGG_PATHWAY	hsa04110	Cell cycle	0.000165791
KEGG_PATHWAY	hsa05214	Glioma	0.00019382
KEGG_PATHWAY	hsa04151	PI3K-Akt signaling pathway	0.000268124
KEGG_PATHWAY	hsa05215	Prostate cancer	0.000306014
KEGG_PATHWAY	hsa04810	Regulation of actin cytoskeleton	0.000309593
KEGG_PATHWAY	hsa04510	Focal adhesion	0.00039945

### 3.4 Screening hub genes in relation to VI

**Figure 4:**
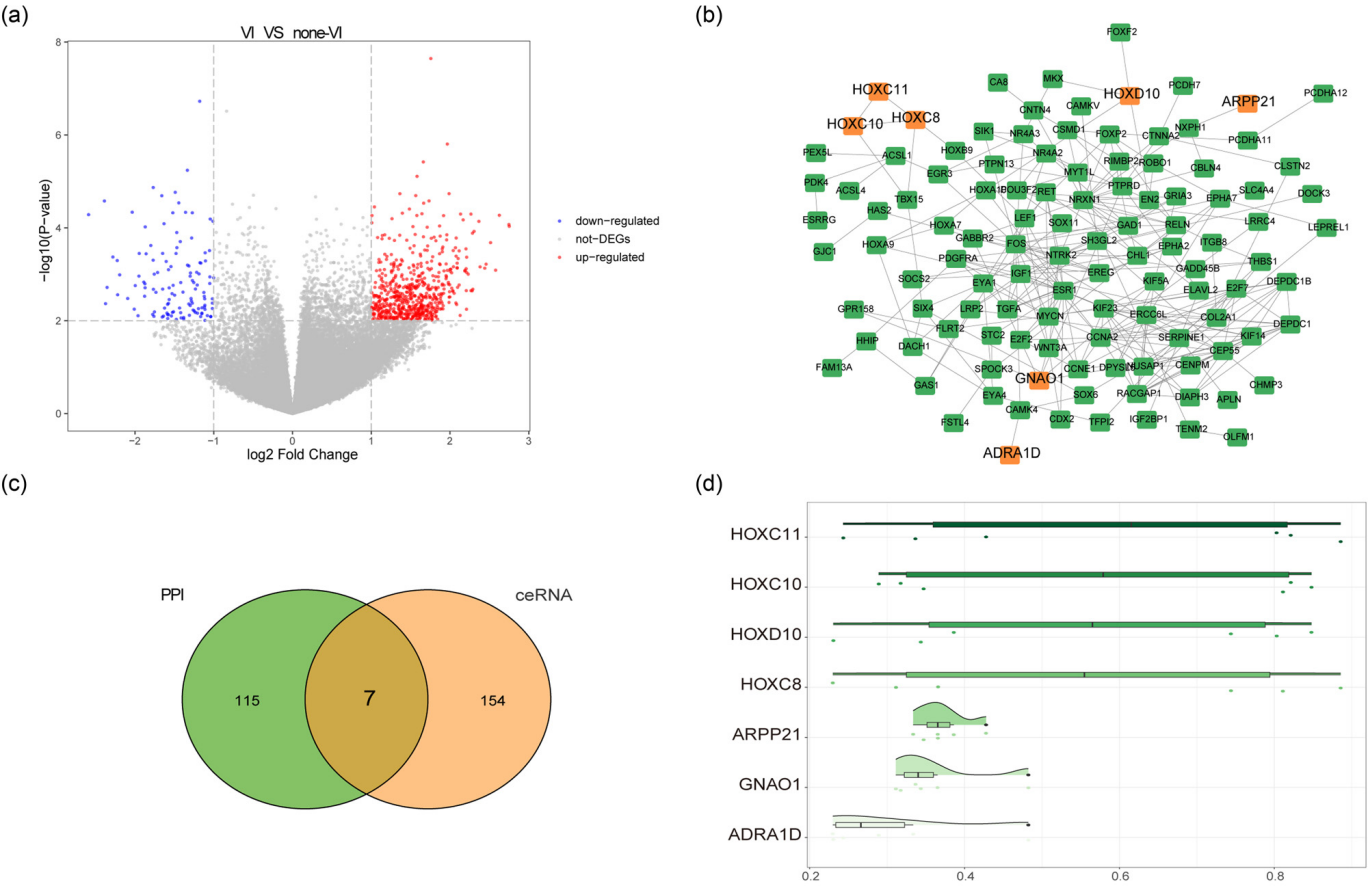
PPI network of DEGs between HCC with and without VI. (a) The DEGs between HCC with and without VI were analyzed, the abscissa was log2 fold change, the ordinate was log10 (adjust *P*-value), the red node was up-regulated DEGs, the blue node was down-regulated DEGs, and the grey node was non-significant DEGs. (b) The PPI network constructed by the encoded proteins of DEGs, and the orange color represents the key genes. (c) The Venn diagram of genes in PPI and DEmRNA in ceRNA network, the orange color represents the DEmRNA in ceRNA network, and green color represents the genes in PPI network. (d) The GO semantic similarity of key genes, the horizontal axis represents similarity, and the vertical axis was the genes.

**Figure A3:**
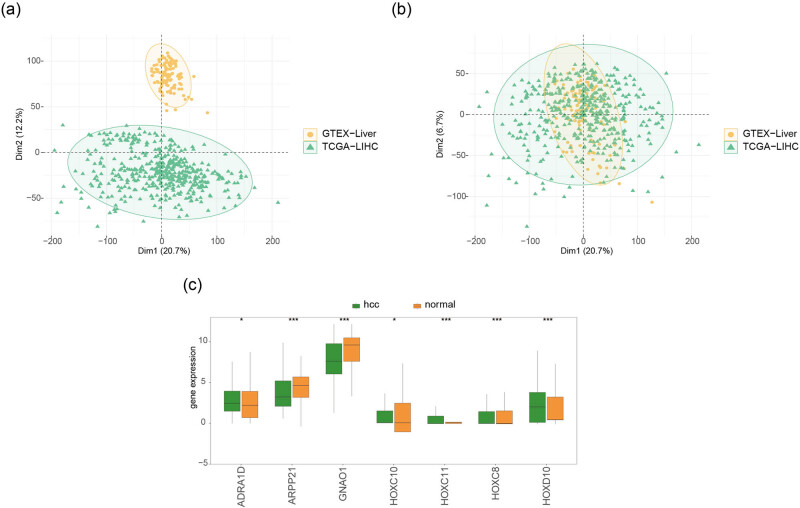
(a) TCGA-LIHC and GTEX-Liver have obvious batch effect. (b) TCGA-LIHC and GTEX-Liver data are integrated after batch effects removal. (c) There was a significant difference in the expression of seven characteristic genes between hepatocellular carcinoma tumor samples and control samples. * it means *P* < 0.05, ** means *P* < 0.01, *** means *P* < 0.001.

A total of 748 DEGs were obtained by differential expression analysis between HCC with VI and those without VI, including 625 up-regulated and 123 down-regulated DEGs (Figure 4a). We screened the proteins encoded by these DEGs to construct a PPI network visualized in Cytoscape (Figure 4b). There are 122 DEGs and 278 PPI pairs in the PPI network. The top five genes that interact with other DEGs are FOS and NRXN1 (interacting with 18 DEGs), CCNA2, ESR1, and NTRK2 (interacting with 17 DEGs). We overlapped the DEGs within the PPI and ceRNA network mentioned above to get seven genes as hub genes (Figure 4c). We used the R packet “GOSemSim” to calculate the GO semantic similarity of these seven hub genes. The results showed that there was a high correlation among HOXC10, HOXC11, HOXC8, and HOXD10 (Figure 4d). Furthermore, the gene expression levels of seven hub genes were screened from two datasets of TCGA-LIHC and GTEX-Liver, and visualized by R-packet ggplot2 box map. The results showed that there were significant differences in the expression of seven hub genes between HCC tumor samples and control samples (Figure A3).

### 3.5 Construction of prognostic risk score model based on hub genes

**Figure 5:**
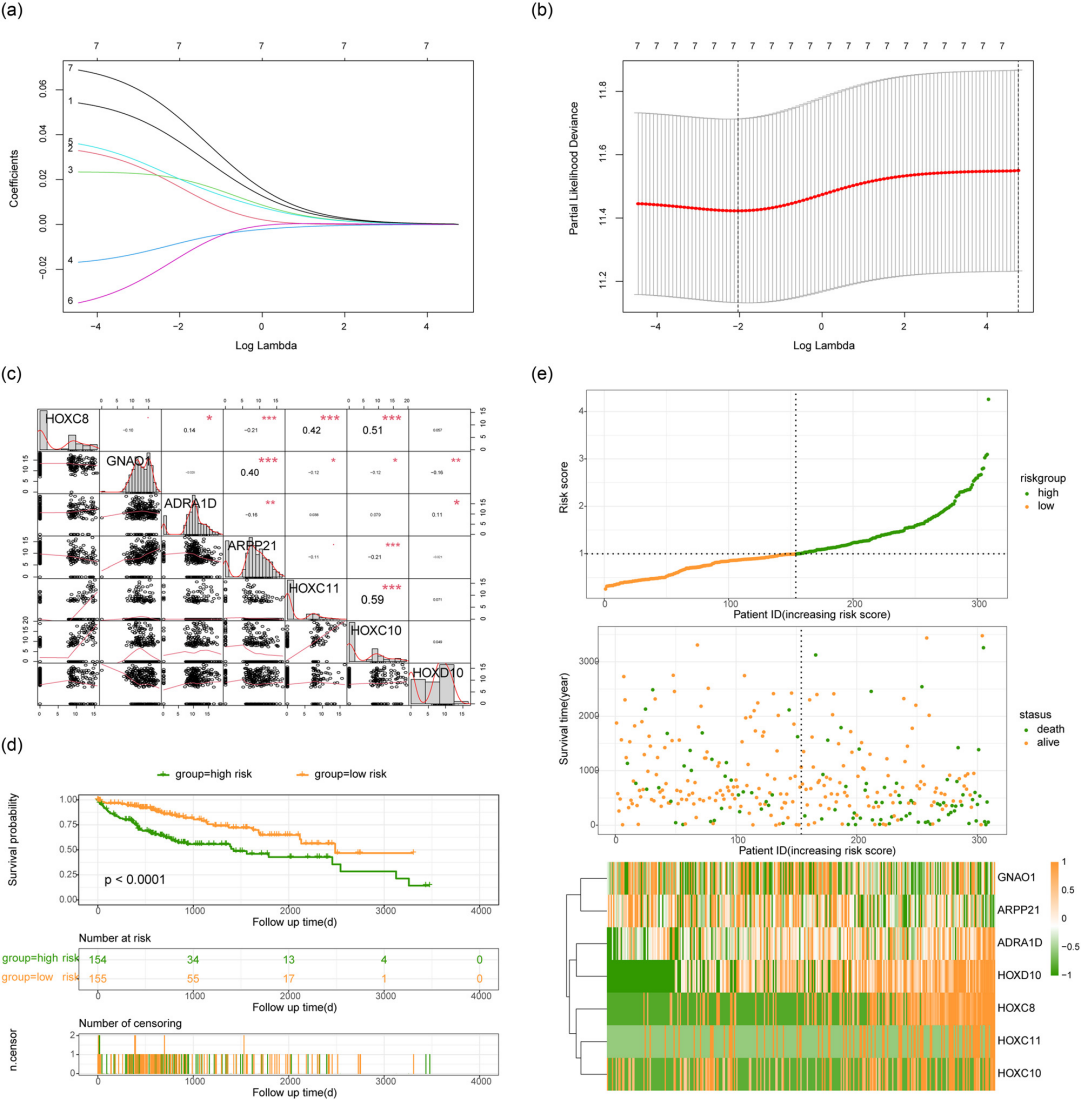
Construction of risk scoring model. (a) LASSO regression characteristic fitting curve. (b) Quantitative analysis of key genes in LASSO regression. (c) Correlation analysis of expression levels of hub genes. (d) Survival analysis between patients in high- and low-risk groups. Orange color indicated patients in low-risk group, green color indicated patients in high-risk group, the horizontal axis was survival time, and the vertical axis was survival probability. (e) Correlation diagram of the risk factors in risk scoring model. The vertical axis in the top figure was the risk score, the horizontal axis was the patient, the vertical axis in the middle figure was the survival time of the patient, and the horizontal axis was the patient. The lower figure is the heat map of the expression levels of key genes, orange color indicats high expression and green color indicates low expression.

We used R packet “glmnet” to obtain the regression coefficients of these seven hub genes based on LASSO method. Combined with the gene expression levels of these hub genes, we constructed a risk score model to predict the prognosis of patients with HCC. The regression coefficients of the seven hub genes were as follows: HOXC8: 0.037, GNAO1: 0.017, ADRA1D: 0.020, ARPP21: −0.008, HOXC11: 0.020, HOXC10: −0.014, and HOXD10: 0.047 (Figure 5a and b). We explored the correlation among the expression of these hub genes, and the results showed high levels of correlation among HOXC10, HOXC11, and HOXC8 (Figure 5c). We calculated the risk score for each patient in the TCGA-HCC cohort, and then divided the patients into high and low risk groups according to the median risk score. KM survival analysis showed that OS in the higher risk group was significantly shorter than that in the lower risk group (*P* < 0.0001, Figure 5d). Next we assessed the relationship between risk score and HCC patient survival. The results showed that the higher the risk score, the shorter the survival time of HCC patient and the higher the proportion of death (Figure 5e).

**Figure A4:**
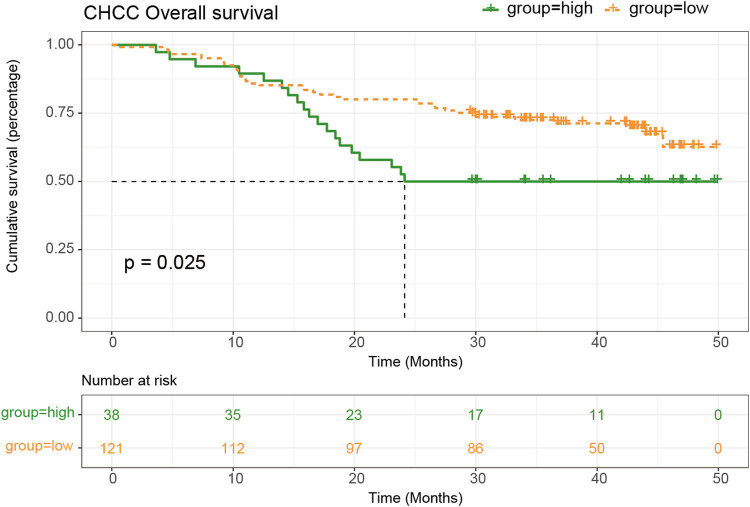
The patients with high risk scores in the validation set of CHCC data also showed a tendency to have a worse prognosis.

In order to verify the robustness of the risk score, we tested the utility of our model in the validation set data. The verification set data were from the Chinese HCC patients with HBV infection (CHCC-HBV) cohort from 2010 to 2014 at Zhongshan Hospital [29], which was accessed through NODE (https://www.biosino.org/node). Combined with the survival data of patients and our model, the risk score of each patient was calculated, and the patients were grouped and analyzed by risk score. The results showed that the patients with high-risk score in the verification set also showed a trend of worse prognosis (Figure A4).

### 3.6 Correlation between risk score and clinical parameters

**Figure 6:**
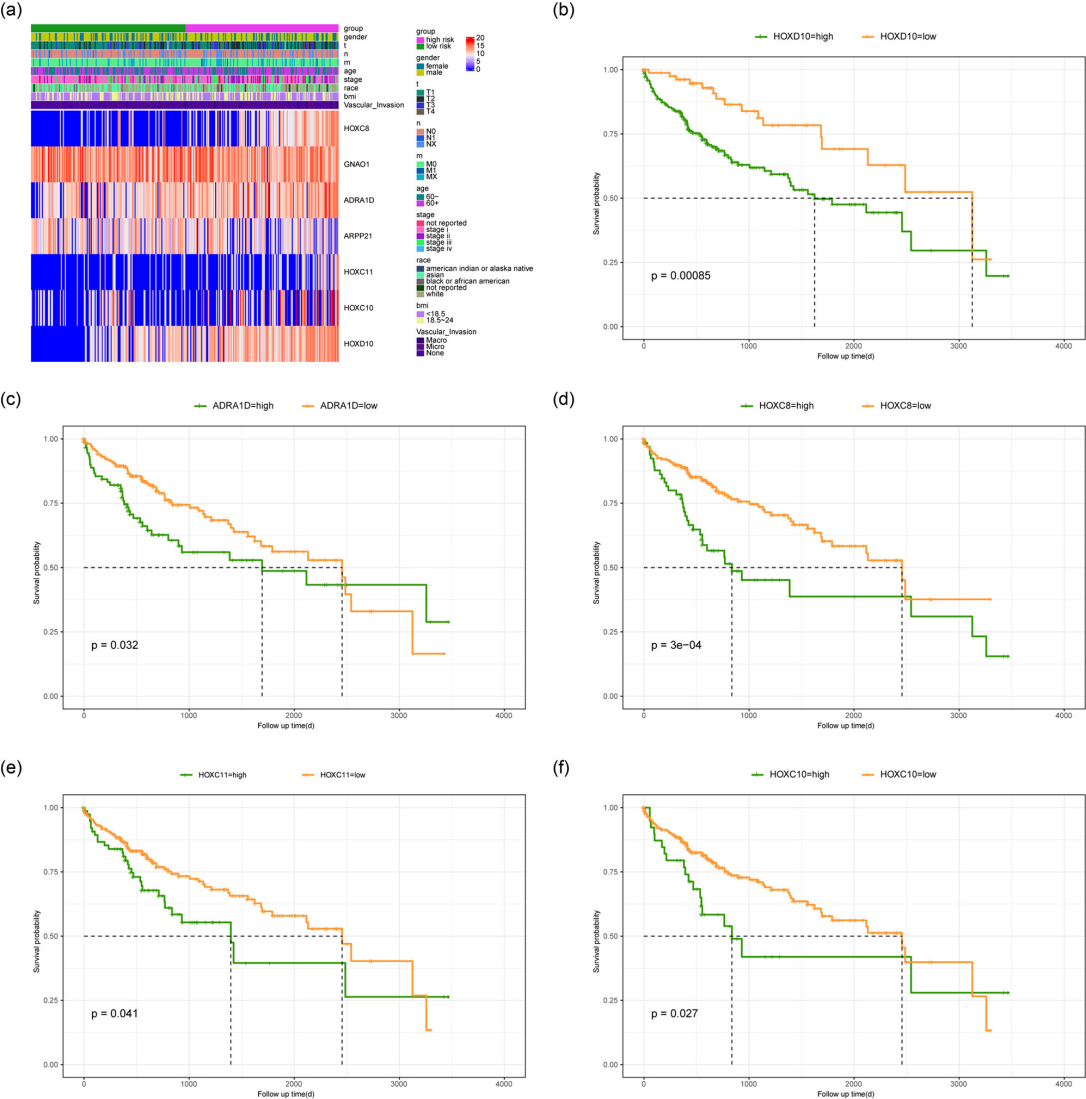
The prognostic relevance of seven characteristic gene. (a) The correlation between risk score, clinical parameter, and key gene expression level. (b)−(f) The survival curves show the effects of HOXC8, ADRA1D, HOXC11, HOXC10, and HOXD10 gene expression on the prognosis of patients.

**Figure 7:**
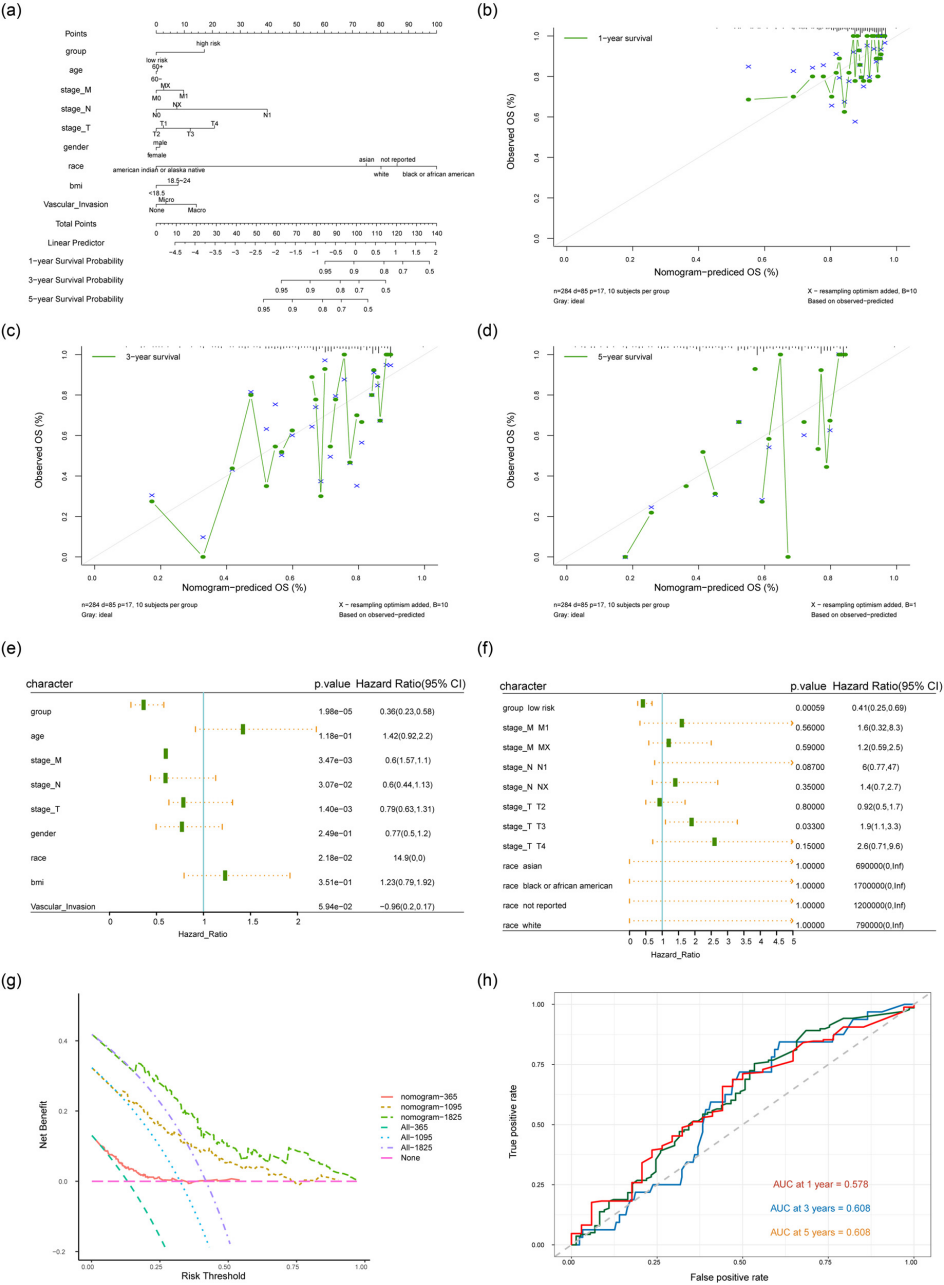
The Nomogram models. (a) Nomograph of HCC patients’ clinical parameters and risk score. (b)–(d) Nomogram model was used to predict the 1 year survival rates, 3 year survival rates, and 5 year survival rates of HCC patients. The horizontal axis and vertical axis were the predicted survival rate and the actual survival rate of HCC patients, respectively. (e) and (f) Univariate and multivariate COX analyses of risk score and HCC patients’ clinical parameters. (g) DCA curve was applied to assess the useful degree of the model for HCC patients. (h) Time ROC analysis was applied to predict 2 year survival rates, 3 year survival rates, and 5 year survival rates of HCC patients.

We analyzed the correlation between risk score and clinical parameters, and the results showed that patients in the high-risk group were usually associated with higher T/N/M stages and VI occurrence (Figure 6a). Then, the effects of the expression levels of seven key genes on the prognosis of patients was analyzed, and the results indicated that the high expression of HOXC8, ADRA1D, HOXC11, HOXC10, and HOXD10 predicted a poor prognosis (Figure 6b−f). In order to assess the effects of risk score and clinical characteristics on the prognosis of HCC patients, the Nomogram model was constructed using clinical parameters and risk score to predict the 1, 3, and 5 year survival rate of patients with HCC. Comparing with the actual 1, 3, and 5 year survival rate of patients, the results showed that the survival rate of HCC patients could be well predicted by the Nomogram model (Figure 7b−d). Univariate Cox analysis using risk score and clinical characteristics showed that risk stratification, T/N/M stage, and ethnicity were all prognostic factors for HCC (Figure 7e). Multivariate Cox analysis showed that risk stratification was the only prognostic factor for HCC (Figure 7f). The DCA curve showed that the model could be beneficial to the prognosis evaluation of patients (Figure 7g). The time-ROC analysis also showed that the 1 year survival prediction performance of the prognostic model was 58%, and both the 3 and 5 year survival prediction performance were 61% (Figure 7h).

### 3.7 Differences in immune infiltration

**Figure 8:**
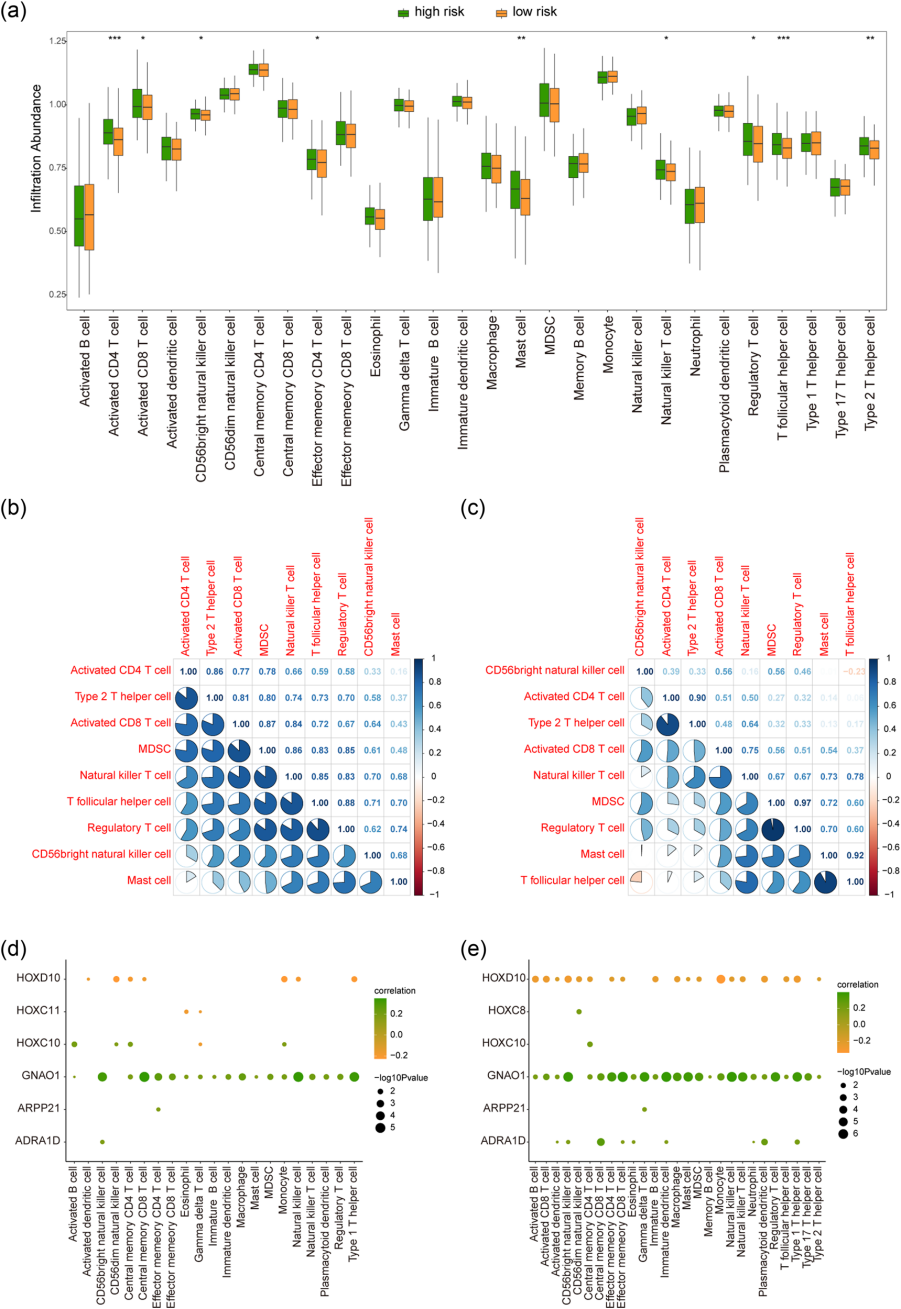
Immune infiltration analysis. (a) The difference of immune cell infiltration between high-risk group and low-risk group, green color indicates high-risk group, orange color indicates low-risk group. (b) and (c) There was a correlation between the immune cell infiltration in the high-risk group and the low-risk group, with blue color indicating a positive correlation and red indicating a negative correlation. (d) and (e) The correlation analysis between immune cell infiltration and hub genes in high-risk group and low-risk group shows that the horizontal axis is immune cell, the vertical axis is key gene, and the node size indicated significant level. The node color represented the correlation level ratio.

After analyzing the immune cell infiltration in HCC, we found that the counts of activated CD4+ T cell, T follicular helper cell, mast cell, type 2T helper cell, activated CD8+ T cell, regulatory T cell, and natural killer T cell in high-risk group were significantly more than that of low-risk group (Figure 8a). Moreover, the correlations between the immune cells content in the high-risk group and the low-risk group were further calculated, and the results showed that the correlation between the immune cells content in the high-risk group was significantly higher than that in the low-risk group (Figure 8b and c). The relationship between hub gene expression and the immune cells of two groups were analyzed and the result showed that there was a noteworthy difference between HOXC11, HOXC8 expression, and immune cells (Figure 8d−e).

## 4 Discussion

Nowadays, HCC is still the leading cause of cancer mortalities globally [30]. Over the past decades, large numbers of studies had been conducted to explore the molecular mechanisms underlying HCC advancement and many target drugs have been applied to treatment, but patients with metastatic HCC still had poor prognosis [31]. Among all the factors, VI stands out due to significant contribution to HCC metastasis, and VI could include angiogenesis and dysfunction of vascular endothelial cell barrier, which provide pathway for cancer cells to invade other organs [32]. Previous study revealed that the presence of microvascular invasion means the presence of tumor invasion to the adjacent tissues of HCC. Microvascular invasion was one of the vital elements of early HCC recurrence and distal metastasis [33]. Therefore, it is of great importance to investigate the molecular mechanism underlying VI of HCC and explore effective treatment target to improve the prognosis of HCC patients.

Increasing evidence has uncovered the significant involvement of noncoding RNAs (ncRNAs) in the tumorigenesis of HCC. circRNAs are covalently closed loop structure ncRNA with evolutionary conservation and high abundance in eukaryotes. Functionally, circRNAs could compete for miRNA binding and remove the inhibitory effect of miRNA on their target mRNAs, thus forming ceRNA mechanism. In addition to functioning as miRNAs sponges, circRNAs could also interact with RNA-binding proteins, and eventually exert regulatory roles in different BPs, including cell proliferation, cell cycle, apoptosis, migration, EMT invasion, glycolysis, angiogenesis, VI, and metastasis [34]. A growing body of studies has demonstrated that circRNAs play important roles in the occurrence and development of HCC and other liver diseases. Most importantly, circRNAs have great clinical value as potential biomarkers and therapeutic target for HCC [35,36]. Therefore, more and more attention has been paid to circRNAs in HCC. Very recently, several circRNA-related ceRNA networks were identified in HCC and subsequently the prognostic risk score models were built based on key genes within these ceRNA network. Han et al. reported a ceRNA network composed of DEcircRNAs and then a prognostic risk assessment model was developed based on seven hub genes (PLOD2, TARS, RNF19B, CCT2, RAN, C5orf30, and MCM10), which was verified to be an independent factor for predicting prognosis of HCC [37]. Notably, two lncRNA-related ceRNA networks associated with VI have been reported in HCC by Cai et al. [38] and Tao et al. [16], respectively, and the prognostic signatures were also built based on distinct DEGs. However, to our knowledge, the prognostic risk score models for HCC based on hub genes in relation to both VI and circRNA-related ceRNAs have never been reported. In the current study, the differences of gene expression between HCC and adjacent normal tissue were explored first, and then we identified a novel circRNA-miRNA-mRNA network consisting of 12 DEmiRNAs, 11 DEcircRNAs, and 161 DEmRNAs. Previous study demonstrated that a circRNA-miRNA-mRNA network involved in the pathogenesis and therapy strategy of HCC [39], and the DEmiRNAs, DEcircRNAs, and DEmRNAs were all different from our study, it might be explained that the distinct database and biological function were investigated.

In our study, the DEGs between HCC with and without VI were also analyzed and used to build a PPI network. The top five genes that interacted with other DEGs were FOS, NRXN1, CCNA2, ESR1, and NTRK2. The great importance of FOS in HCC has also been reported by others. Study has verified that FOS might be a potential marker for predicting HCC prognostic. The expression level of FOS in HCC patients was inversely related to the OS [40]. Furthermore, we made the intersection of the DEGs within the PPI network and the DEGs in the ceRNA network to get seven hub genes. Subsequently, a seven-gene based prognostic risk score model was constructed for HCC. The result of TCGA-HCC cohort showed that the OS of the higher risk group was markedly shorter than that of the lower risk group. These seven hub genes were GNAO1, ADRA1D, ARPP21, HOXC8, HOXC11, HOXC10, and HOXD10. Some of these hub genes have been reported to be implicated in HCC progression. GNAO1 was shown to be significantly downregulated in HCC, as well as being implicated in a variety of intracellular biological events. GNAO1 may act as a tumor suppressor and was a reliable biomarker of relapse prediction for HCC [41,42]. A previous bioinformatics analysis revealed that HOXC genes (HOXC8, HOXC9, HOXC10, HOXC11, HOXC12, and HOXC13) might participate in pathogenesis of gastric adenocarcinoma [43]. In addition, high expression levels of HOXC genes were significantly correlated with shorter OS of the gastric cancer patients [44]. Here we found that HOX genes might play the same roles in HCC, as KM analysis demonstrated that HOXC8, HOXC11, HOXC10, and HOXD10 expressions were all inversely related to HCC patients’ OS. To our knowledge, this is the first time to report the expression and the prognostic role of HOXC genes in HCC.

It is generally believed that HCC is preceded by liver damage and extensive inflammation, and therefore is accompanied by immune cells infiltration. Intratumoral immune cell infiltration has been associated with HCC prognosis in previous study [45]. HCC is often accompanied with a dense stroma coupled with infiltrated immune cells, referring to as the tumor microenvironment. Populations of infiltrated immune cells, such as CD163+ macrophages and CD8+ T cells, are associated with the prognosis in HCC, and immune cells in the tumor microenvironment can be a target for HCC therapy [46]. Here we analyzed the immune cell infiltration in HCC patients according to the risk stratification based on the prognostic model. The counts of several important immune cells, such as Activated CD4+ T cell, Mast cell, Activated CD8+ T cell, and Natural killer T, in high-risk group were all significantly higher than those in low-risk group, and the correlation of infiltrated immune cells in high-risk group was also significantly higher than that in low-risk group. This indicated that our prognostic model was useful in predicting immune response in HCC. Moreover, our data showed a significant correlation among the expression levels of HOXC11, HOXC8, and immune cells infiltration in HCC, indicating that the HOXC genes might play vital roles in immune response of HCC.

It should be noticed that there are some limits in our study. First, the data sources and any assumptions inherent in the analysis process may enhance the potential bias of the results of this study; therefore, larger sample sizes, more databases, and better algorithms are needed to build a more comprehensive ceRNA network in HCC. Second, HCC tissue sample detection in our research center should be performed to test the clinical utility of the prognostic model. Furthermore, the role of HOXC genes in HCC progression should be verified *in vitro* and *in vivo* experiments, which might explore the underling molecular mechanism for HCC metastasis.

Furthermore, we have also noticed the technical limitations and uncertainties in applying our model to real-world scenarios. Liver cancer is a complex disease with various unknown factors and variables influencing its progression and treatment. Therefore, our model’s predictions may carry a certain degree of uncertainty, particularly in complex clinical cases. We believe it is crucial to highlight this aspect to ensure transparent communication and informed decision-making by healthcare professionals.

## 5 Conclusion

Here we established a novel prognostic model based on seven hub genes, which were screened from the intersection of DEGs within a VI-related PPI network and a circRNA-related ceRNA network. The seven-gene based prognostic model was useful for evaluating the prognosis of HCC patients. This study clarified for the first time that the abnormal expression and the prognostic roles of HOXC genes (HOXC10, HOXC11, and HOXC8) in HCC. Additionally, the expression of HOXC11 and HOXC8 were correlated with immune cell infiltration, suggesting that they might be potential immunotherapy targets for HCC. Further studies are needed to verify these results *in vitro* and *in vivo*.

## References

[1] McGlynn KA, Petrick JL, El-Serag HB. Epidemiology of hepatocellular carcinoma. Hepatology. 2021;73(Suppl 1):4–13. 10.1002/hep.31288 PMC7577946 32319693

[2] Ganesan P, Kulik LM. Hepatocellular carcinoma: New developments. Clliver Dis. 2023;27:85–102. 10.1016/j.cld.2022.08.004 36400469

[3] Brown ZJ, Tsilimigras DI, Ruff SM, Mohseni A, Kamel IR, Cloyd JM, et al. Management of hepatocellular carcinoma: A review. JAMA Surg. 2023;158:410–20. 10.1001/jamasurg.2022.7989 36790767

[4] Chidambaranathan-Reghupaty S, Fisher PB, Sarkar D. Hepatocellular carcinoma (HCC): Epidemiology, etiology and molecular classification. Adv Cancer Res. 2021;149:1–61. 10.1016/bs.acr.2020.10.001 PMC8796122 33579421

[5] Isik B, Gonultas F, Sahin T, Yilmaz S. Microvascular venous invasion in hepatocellular carcinoma: Why do recurrences occur? J Gastrointest Cancer. 2020;51:1133–6. 10.1007/s12029-020-00487-9 32839943

[6] Sulaiman SA, Abu N, Ab-Mutalib NS, Low TY, Jamal R. Signatures of gene expression, DNA methylation and microRNAs of hepatocellular carcinoma with vascular invasion. Future Oncol (London, Engl). 2019;15:2603–17. 10.2217/fon-2018-0909 31339048

[7] Lin Z, Cai YJ, Chen RC, Chen BC, Zhao L, Xu SH, et al. A microRNA expression profile for vascular invasion can predict overall survival in hepatocellular carcinoma. Clinica Chim Acta; Int J Clin Chem. 2017;469:171–9. 10.1016/j.cca.2017.03.026 28365450

[8] Pang RW, Joh JW, Johnson PJ, Monden M, Pawlik TM, Poon RT. Biology of hepatocellular carcinoma. Ann Surgical Oncol. 2008;15:962–71. 10.1245/s10434-007-9730-z 18236113

[9] Zhu LP, He YJ, Hou JC, Chen X, Zhou SY, Yang SJ, et al. The role of circRNAs in cancers. Biosci Rep. 2017;37(5):BSR20170750. 10.1042/BSR20170750 PMC5653918 28928231

[10] Chen J, Qi Z. The elevated circ_0067835 could accelerate cell proliferation and metastasis via miR-1236-3p/Twist2 axis in hepatocellular carcinoma. BioMed Res Int. 2022;2022:2825172. 10.1155/2022/2825172 PMC9576392 36262967

[11] Zhou Z, Cui X, Gao P, Zhang X, Zhu C, Sun B. Circular RNA circRASSF5 functions as an anti-oncogenic factor in hepatocellular carcinoma by acting as a competitive endogenous RNA through sponging miR-331-3p. J Hepatocell Carcinoma. 2022;9:1041–56. 10.2147/JHC.S376063 PMC9547604 36217445

[12] Song LN, Qiao GL, Yu J, Yang CM, Chen Y, Deng ZF, et al. Hsa_circ_0003998 promotes epithelial to mesenchymal transition of hepatocellular carcinoma by sponging miR-143-3p and PCBP1. J Exp Clin Cancer Res CR. 2020;39:114. 10.1186/s13046-020-01576-0 PMC7302140 32552766

[13] Chen R, Chen Y, Huang W, Zhao Y, Luo W, Lin J, et al. Comprehensive analysis of an immune-related ceRNA network in identifying a novel lncRNA signature as a prognostic biomarker for hepatocellular carcinoma. Aging. 2021;13:17607–28. 10.18632/aging.203250 PMC8312417 34237706

[14] Zhang Q, Sun L, Zhang Q, Zhang W, Tian W, Liu M, et al. Construction of a disease-specific lncRNA-miRNA-mRNA regulatory network reveals potential regulatory axes and prognostic biomarkers for hepatocellular carcinoma. Cancer Med. 2020;9:9219–35. 10.1002/cam4.3526 PMC7774738 33232580

[15] Huang K, Lu Z, Li L, Peng G, Zhou W, Ye Q. Construction of a ceRNA network and a genomic-clinicopathologic nomogram to predict survival for HBV-related HCC. Hum Cell. 2021;34:1830–42. 10.1007/s13577-021-00607-y 34487338

[16] Tao H, Li J, Liu J, Yuan T, Zhang E, Liang H, et al. Construction of a ceRNA network and a prognostic lncRNA signature associated with vascular invasion in hepatocellular carcinoma based on weighted gene co-expression network analysis. J Cancer. 2021;12:3754–68. 10.7150/jca.57260 PMC8176257 34093785

[17] Fu L, Yao T, Chen Q, Mo X, Hu Y, Guo J. Screening differential circular RNA expression profiles reveals hsa_circ_0004018 is associated with hepatocellular carcinoma. Oncotarget. 2017;8:58405–16. 10.18632/oncotarget.16881 PMC5601662 28938566

[18] Han D, Li J, Wang H, Su X, Hou J, Gu Y, et al. Circular RNA circMTO1 acts as the sponge of microRNA-9 to suppress hepatocellular carcinoma progression. Hepatology. 2017;66:1151–64. 10.1002/hep.29270 28520103

[19] Chakraborty S, Datta S, Datta S. Surrogate variable analysis using partial least squares (SVA-PLS) in gene expression studies. Bioinformatics. 2012;28:799–806. 10.1093/bioinformatics/bts022 22238271

[20] Szklarczyk D, Gable AL, Lyon D, Junge A, Wyder S, Huerta-Cepas J, et al. STRING v11: protein-protein association networks with increased coverage, supporting functional discovery in genome-wide experimental datasets. Nucleic Acids Res. 2019;47:D607–13. 10.1093/nar/gky1131 PMC6323986 30476243

[21] Ritchie ME, Phipson B, Wu D, Hu Y, Law CW, Shi W, et al. Limma powers differential expression analyses for RNA-sequencing and microarray studies. Nucleic Acids Res. 2015;43:e47. 10.1093/nar/gkv007 PMC4402510 25605792

[22] Kolde R, Kolde MR. Package ‘pheatmap’. R package. 2015;1:790.

[23] Ashburner M, Ball CA, Blake JA, Botstein D, Butler H, Cherry JM, et al. Gene ontology: tool for the unification of biology. The gene ontology consortium. Nat Genet. 2000;25:25–9. 10.1038/75556 PMC3037419 10802651

[24] Kanehisa M, Goto S. KEGG: kyoto encyclopedia of genes and genomes. Nucleic Acids Res. 2000;28:27–30. 10.1093/nar/28.1.27 PMC102409 10592173

[25] Yu G, Wang LG, Han Y, He QY. ClusterProfiler: an R package for comparing biological themes among gene clusters. OMICS. 2012;16:284–7. 10.1089/omi.2011.0118 PMC3339379 22455463

[26] Engebretsen S, Bohlin J. Statistical predictions with glmnet. Clin Epigenetics. 2019;11:123. 10.1186/s13148-019-0730-1 PMC6708235 31443682

[27] Nunez E, Steyerberg EW, Nunez J. [Regression modeling strategies]. Rev Esp Cardiol. 2011;64:501–7. 10.1016/j.recesp.2011.01.019 21531065

[28] Barbie DA, Tamayo P, Boehm JS, Kim SY, Moody SE, Dunn IF, et al. Systematic RNA interference reveals that oncogenic KRAS-driven cancers require TBK1. Nature. 2009;462:108–12. 10.1038/nature08460 PMC2783335 19847166

[29] Gao Q, Zhu H, Dong L, Shi W, Chen R, Song Z, et al. Integrated Proteogenomic Characterization of HBV-Related Hepatocellular Carcinoma. Cell. 2019;179(561−577):e22. 10.1016/j.cell.2019.08.052 31585088

[30] Huang XY, Huang ZL, Huang J, Xu B, Huang XY, Xu YH, et al. Exosomal circRNA-100338 promotes hepatocellular carcinoma metastasis via enhancing invasiveness and angiogenesis. J Exp Clin Cancer Res CR. 2020;39:20. 10.1186/s13046-020-1529-9 PMC6979009 31973767

[31] Yu J, Xu QG, Wang ZG, Yang Y, Zhang L, Ma JZ, et al. Circular RNA cSMARCA5 inhibits growth and metastasis in hepatocellular carcinoma. J Hepatol. 2018;68:1214–27. 10.1016/j.jhep.2018.01.012 29378234

[32] Wang W, Guo Y, Zhong J, Wang Q, Wang X, Wei H, et al. The clinical significance of microvascular invasion in the surgical planning and postoperative sequential treatment in hepatocellular carcinoma. Sci Rep. 2021;11:2415. 10.1038/s41598-021-82058-x PMC7843639 33510294

[33] Erstad DJ, Tanabe KK. Prognostic and therapeutic implications of microvascular invasion in hepatocellular carcinoma. Ann Surg Oncol. 2019;26:1474–93. 10.1245/s10434-019-07227-9 30788629

[34] Niu ZS, Wang WH. Circular RNAs in hepatocellular carcinoma: Recent advances. World J Gastrointest Oncol. 2022;14:1067–85. 10.4251/wjgo.v14.i6.1067 PMC9244981 35949213

[35] Meng H, Niu R, Huang C, Li J. Circular RNA as a novel biomarker and therapeutic target for HCC. Cells. 2022;11(12):1948. 10.3390/cells11121948 PMC9222032 35741077

[36] Chen T. Circulating Non-Coding RNAs as potential diagnostic biomarkers in hepatocellular carcinoma. J Hepatocell Carcinoma. 2022;9:1029–40. 10.2147/JHC.S380237 PMC9484560 36132427

[37] Han L, Wang M, Yang Y, Xu H, Wei L, Huang X. Detection of Prognostic Biomarkers for Hepatocellular Carcinoma through CircRNA-associated CeRNA Analysis. J Clin Transl Hepatol. 2022;10:80–9. 10.14218/JCTH.2020.00144 PMC8845162 35233376

[38] Cai S, Du R, Zhang Y, Yuan Z, Shang J, Yang Y, et al. Construction and comprehensive analysis of ceRNA networks and tumor-infiltrating immune cells in hepatocellular carcinoma with vascular invasion. Front Bioinforma. 2022;2:836981. 10.3389/fbinf.2022.836981 PMC9580849 36304284

[39] Xiong DD, Dang YW, Lin P, Wen DY, He RQ, Luo DZ, et al. A circRNA-miRNA-mRNA network identification for exploring underlying pathogenesis and therapy strategy of hepatocellular carcinoma. J Transl Med. 2018;16:220. 10.1186/s12967-018-1593-5 PMC6085698 30092792

[40] Hu JW, Ding GY, Fu PY, Tang WG, Sun QM, Zhu XD, et al. Identification of FOS as a candidate risk gene for liver cancer by integrated bioinformatic analysis. BioMed Res Int. 2020;2020:6784138. 10.1155/2020/6784138 PMC7125454 32280695

[41] Pei X, Zhang J, Wu L, Lü B, Zhang X, Yang D, et al. The down-regulation of GNAO1 and its promoting role in hepatocellular carcinoma. Biosci Rep. 2013;33(5):e00069. 10.1042/BSR20130001 PMC3775511 23984917

[42] Du M, Feng J, Tao Y, Pan Q, Chen F. GNAO1 as a Novel Predictive Biomarker for Late Relapse in Hepatocellular Carcinoma. J Healthc Eng. 2021;2021:7631815. 10.1155/2021/7631815 PMC8654523 34900204

[43] Fu T, Ji X, Bu Z, Zhang J, Wu X, Zong X, et al. Identification of key long non-coding RNAs in gastric adenocarcinoma. Cancer Biomarkers: Sect A Dis Markers. 2020;27:541–53. 10.3233/CBM-192389 PMC12662306 32176636

[44] Gu H, Zhong Y, Liu J, Shen Q, Wei R, Zhu H, et al. The role of miR-4256/HOXC8 signaling axis in the gastric cancer progression: Evidence from lncRNA-miRNA-mRNA network analysis. Front Oncol. 2021;11:793678. 10.3389/fonc.2021.793678 PMC8801578 35111675

[45] Chew V, Chen J, Lee D, Loh E, Lee J, Lim KH, et al. Chemokine-driven lymphocyte infiltration: an early intratumoural event determining long-term survival in resectable hepatocellular carcinoma. Gut. 2012;61:427–38. 10.1136/gutjnl-2011-300509 PMC3273680 21930732

[46] Pham L, Kyritsi K, Zhou T, Ceci L, Baiocchi L, Kennedy L, et al. The functional roles of immune cells in primary liver cancer. Am J Pathol. 2022;192:826–36. 10.1016/j.ajpath.2022.02.004 PMC9194651 35337836

